# smplot: An R Package for Easy and Elegant Data Visualization

**DOI:** 10.3389/fgene.2021.802894

**Published:** 2021-12-15

**Authors:** Seung Hyun Min, Jiawei Zhou

**Affiliations:** School of Ophthalmology and Optometry, Affiliated Eye Hospital, State Key Laboratory of Ophthalmology, Optometry and Vision Science, Wenzhou Medical University, Wenzhou, China

**Keywords:** smplot, data visualisation, R software, data analysis, ggplot2

## Abstract

R, a programming language, is an attractive tool for data visualization because it is free and open source. However, learning R can be intimidating and cumbersome for many. In this report, we introduce an R package called “smplot” for easy and elegant data visualization. The R package “smplot” generates graphs with defaults that are visually pleasing and informative. Although it requires basic knowledge of R and ggplot2, it significantly simplifies the process of plotting a bar graph, a violin plot, a correlation plot, a slope chart, a Bland-Altman plot and a raincloud plot. The aesthetics of the plots generated from the package are elegant, highly customisable and adhere to important practices of data visualization. The functions from smplot can be used in a modular fashion, thereby allowing the user to further customise the aesthetics. The *smplot* package is open source under the MIT license and available on Github (https://github.com/smin95/smplot), where updates will be posted. All the example figures in this report are reproducible and the codes and data are provided for the reader in a separate online guide (https://smin95.github.io/dataviz/).

## Introduction

Data visualization is an important skill in scientific writing. The reader may agree that most memorable aspects of a scientific paper are its figures rather than texts. There are various programs for plotting data. However, some require subscription fees, such as Matlab. On the other hand, others such as matplotlib in Python (Hunter, 2007) and *ggplot2* in R (Wickham, 2016) are free and open source but can overwhelm incoming research trainees because the students are often required to overcome a steep learning curve. Moreover, the learning curves can enforce students to spend a long time to change typesetting or making such minute changes, forcing them to use vector graphics editor such as Adobe Illustrator to polish the figures instead of modifying the codes that generate the original plot. This practice of creating a figure using multiple programs, however, can be time-consuming in the long run. For instance, when the trainee is asked to make changes in the figure, one must make changes in all programs that one has used sequentially, which can be tedious and laborious. In this report, we hope to convince the reader that a polished, satisfying figure can be created using only one software environment by introducing a new, free, and easy-to-use tool for data visualization.

Biomedical research increasingly incorporates the usage of complex, computational tools for data analysis. For this reason, we introduce an R package “smplot” that is an intuitive and quick tool for performing elegant data visualization for research trainees. Since the use of smplot requires a basic knowledge of R and ggplot2, an online tutorial about R that incorporates smplot has been posted on a separate webpage entitled *Data Visualization in R Using smplot* (https://smin95.github.io/dataviz).

### Why R?

In R, one can plot data without necessarily using programming concepts such as the *for loop*. This is because the *ggplot2* package in R can automatically plot all data points if necessary. However, this is not the case with Python (matplotlib) and Matlab. All the codes and data for the figures in this report can be found in Chapter 6 of the online guide entitled *Data Visualization in R Using smplot* (https://smin95.github.io/dataviz).

## Methods

### Installation of the smplot Package

At the time of writing the paper, the smplot package is only available on Github. Therefore, if the reader is interested in installing the package, the reader must open RStudio and directly download the package by typing these commands:

install.packages (‘devtools).

devtools:install_github (“smin95/smplot”)

To load the smplot package into the local environment (and therefore use it), the reader must type this code below:

library (smplot).

A complete tutorial on *smplot* is available in Chapter 4 of the online guide (https://smin95.github.io/dataviz/). If the reader is not familiar with *R*, then please consider reading the online guide from Chapter 1 (https://smin95.github.io/dataviz/download-rstudio-basics-of-r.html). If the reader is familiar with ggplot2 and only interested in recreating the figures in this report, please read Chapter 6 (https://smin95.github.io/dataviz/recreating-the-manuscript-figures.html). The package is scheduled to be submitted to the CRAN (The Comprehensive R Archive Network) in near future. All updates will be posted on Github and the online guide.

## Results and Discussion

### Correlation Plot

A correlation refers to a relationship between two variables. The smplot package provides some functions for plotting a correlation.

**FIGURE 1:**
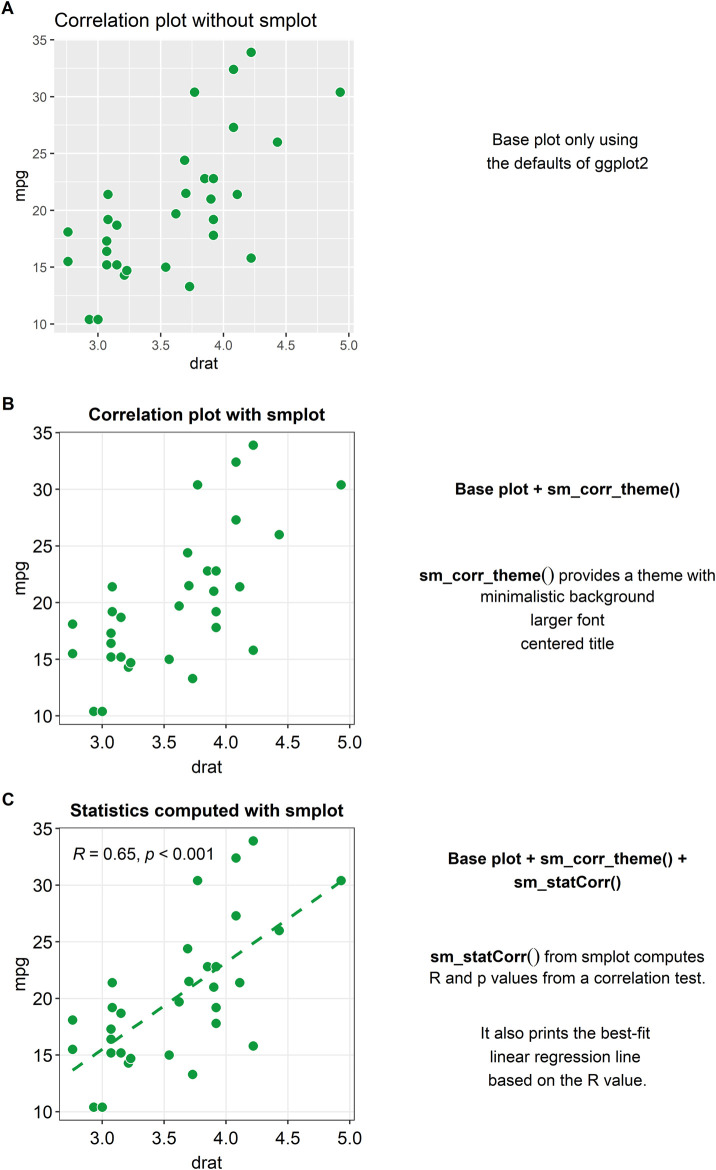
Correlation plots with and without *smplot*. **(A)** A correlation plot without using *smplot*. **(B)** A correlation plot with a default theme of *smplot*. The theme can added in a modular fashion by adding “*sm_corr_theme()*” to the base plot. This function provides a theme with a minimalistic background, a larger font and a centered title. **(C)** A correlation plot with the default theme of *smplot*, printed statistical information (*R* = correlation coefficient, p = statistical significance) and a best-fit regression line. The *R* and *p* values as well as the regression line can be printed by adding the “*sm_statCorr()*” to the given plot in a modular fashion.

Figure 1A shows a correlation plot with defaults of ggplot2 and without smplot. The example is cluttered with distracting features, such as the grey background, and major and minor vertical and horizontal grid lines. Also, the title is not centered. These issues can be resolved by modularly adding a single line of code provided by *smplot*, as shown in Figure 1B. The example in Figure 1B uses the default theme of *smplot* [with the function “*sm_corr_theme()*”]. The minor grid lines have been removed and the title has been centered. Also, the font is generally larger and consistent. The aesthetics can be modified by adding the ggplot2 functions to the base plot. However, *smplot* provides a wrapper function for a clean default theme that can be added in a modular fashion to the base plot. This modularity can allow the user to customise further with ease.

When plotting a correlation, one is often recommended to report statistics and print a best-fit regression line. A function called “sm_statCorr()” can be added modularly to print the correlation coefficient (R, not R^2^) and the *p*-value for statistical significance of the relationship between two variables (see Figure 1C). There are several arguments that are used in this function. The regression is set to be linear by default but can also be set to be non-linear by specifying the argument “*lm_method*” (ex., “*lm_method = lm*” for linear regression, “*lm_method = loess*” for non-linear local regression). Also, the type of the correlation test can be specified into either Pearson, Spearman or Kendall using the argument “*corr_method*” (ex., “*corr_method = pearson*” is the default). When the user adds “sm_statCorr()” modularly to the base plot without specifying these arguments, the function uses the defaults for the two arguments.

### Bar Plot

Plotting a bar graph in *ggplot2* can appear to be not straightforward because the functions that plot the bar graph depend on the structure of the data file that is uploaded in RStudio. For instance, although both “*geom_bar()*” and “*stat_summary()*,” which has multiple usages, can both plot the bar graph in a *ggplot2* setting, “*geom_bar()*” requires that the loaded data contain summarised data (ex., mean, standard error of the sample), whereas “*stat_summary()*” requires that the loaded data contain individual data so that function can directly summarise the data as the mean and the standard deviation. This subtle difference between the functions can be confusing. Also, the arguments for the function “*stat_summary()*” are not always clear.

**FIGURE 2:**
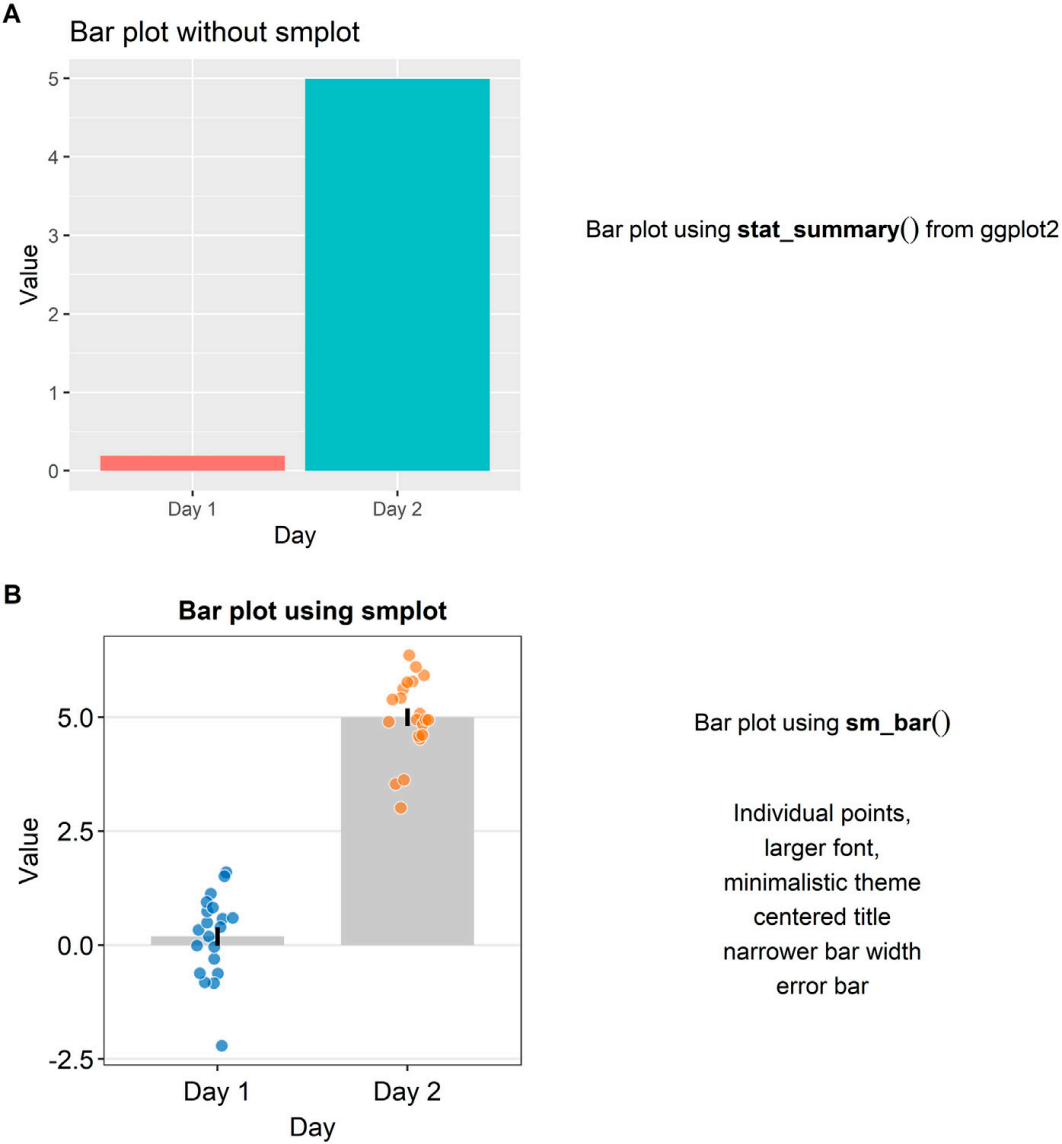
Bar plots with and without *smplot*. **(A)** A bar plot drawn with “*stat_summary()*”, which is a function of ggplot2. **(B)** A bar plot drawn with “*sm_bar()*”, which is a function of smplot. This function automatically provides several features, such as individual data points, larger font, minimalistic theme, centered title, narrower bar width and error bars, such as standard error, standard deviation or 95% confidence interval.

In Figure 2A, a bar graph that uses the default theme of *ggplot2* is shown. Individual data points and error bars are missing. Major and minor vertical and horizontal grids overly crowd the graph. In a bar graph, since explanatory variables (levels in the x-axis) are often categorical, vertical grids are often not necessary. Also, the bar graph alone does not represent the distribution of data accurately, so plotting individual points and the error bar (ex., standard error, standard deviation or 95% confidence interval) are often recommended when the bar graph is plotted. These issues in Figure 2A can be resolved by modularly adding the function “*sm_bar()*” to the base plot, as shown in Figure 2B. This function enlarges the font, plots individual data points, automatically removes unnecessary grids, centers the title, narrows the bar width for aesthetics, and plots the error bar (in this example: standard error). These aesthetic features, such as the transparency, color and shape of the points, can be customised by using the specifying the arguments of “*sm_bar()*,” such as “*bar_alpha*,” “*point_alpha*” and “*point_size.*” If the reader is interested in learning more about the function, please visit Chapter 4 of the online guide (smin95.github.io/dataviz/).

### Boxplot

A preferred method of illustration to a bar graph when reporting data across different groups/time is a boxplot. It reports the median, 25 and 75% quartiles, spread of the data, distribution and outliers, all of which the bar graph does not show. For instance, the minimum and maximum data points are depicted with the *whiskers* that extend to the top and the bottom of the box in the center. The horizontal line within the box represents the *sample median*. Points that are residing above or below the whiskers represent *outliers*.

**FIGURE 3:**
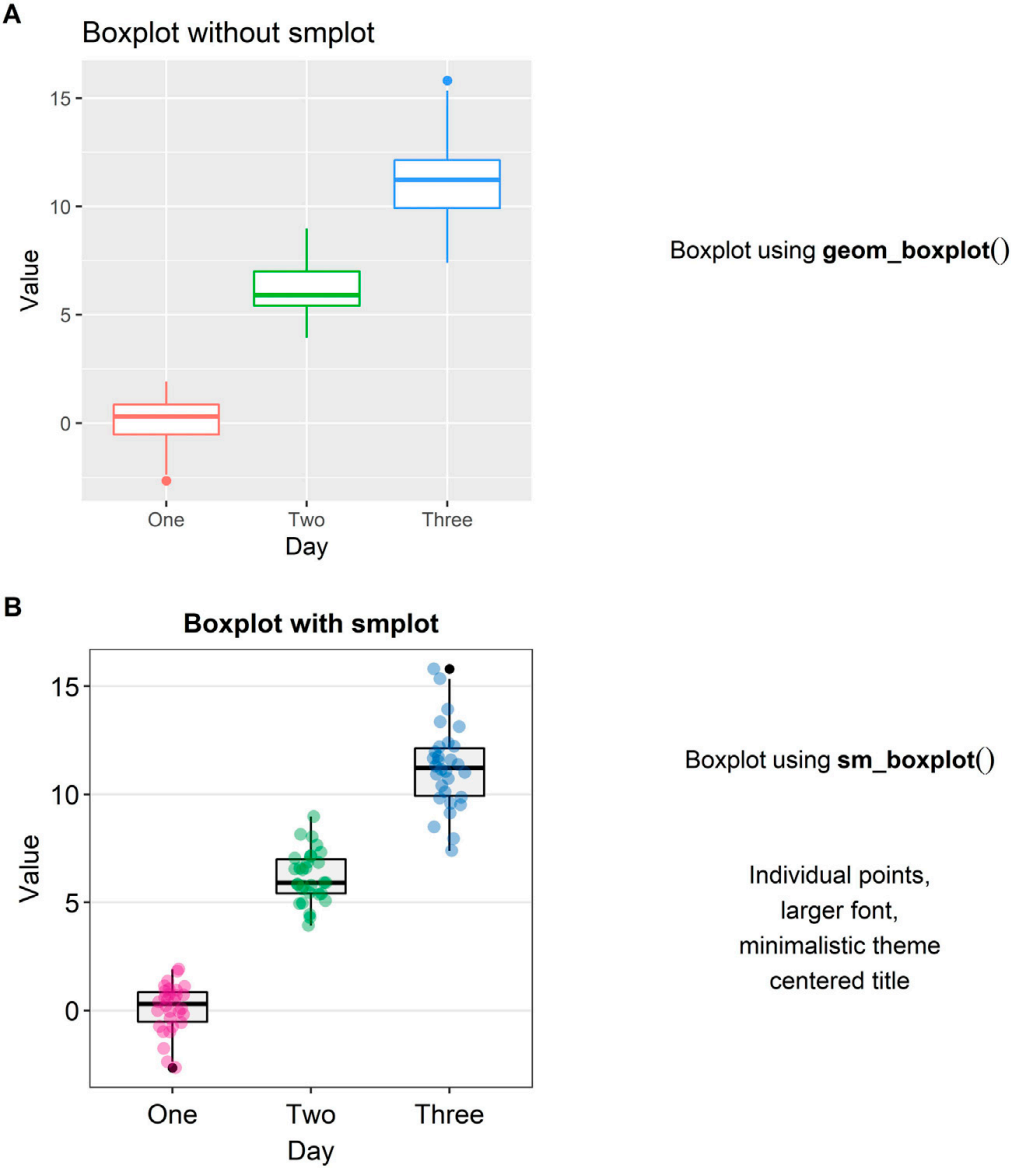
Boxplots with and without *smplot*. **(A)** A boxplot drawn with “*geom_boxplot()*”, which is a function of *ggplot2*. **(B)** A boxplot drawn with “*sm_boxplot()*”. This function automatically provides several features, such as individual data points, larger font, minimalistic theme, and centered title.

In Figure 3A, a boxplot using the default themes of *ggplot2* is shown. On its own, it is not very informative because the individual points are not displayed. Aesthetically, there are some distracting features such as the major and minor vertical and horizontal grids and unnecessarily wide boxes. If the reader adds “*sm_boxplot()*” to the given plot in Figure 3A, she will be able to resolve these aesthetic issues (see Figure 3B).

### Violin Plot

**FIGURE 4:**
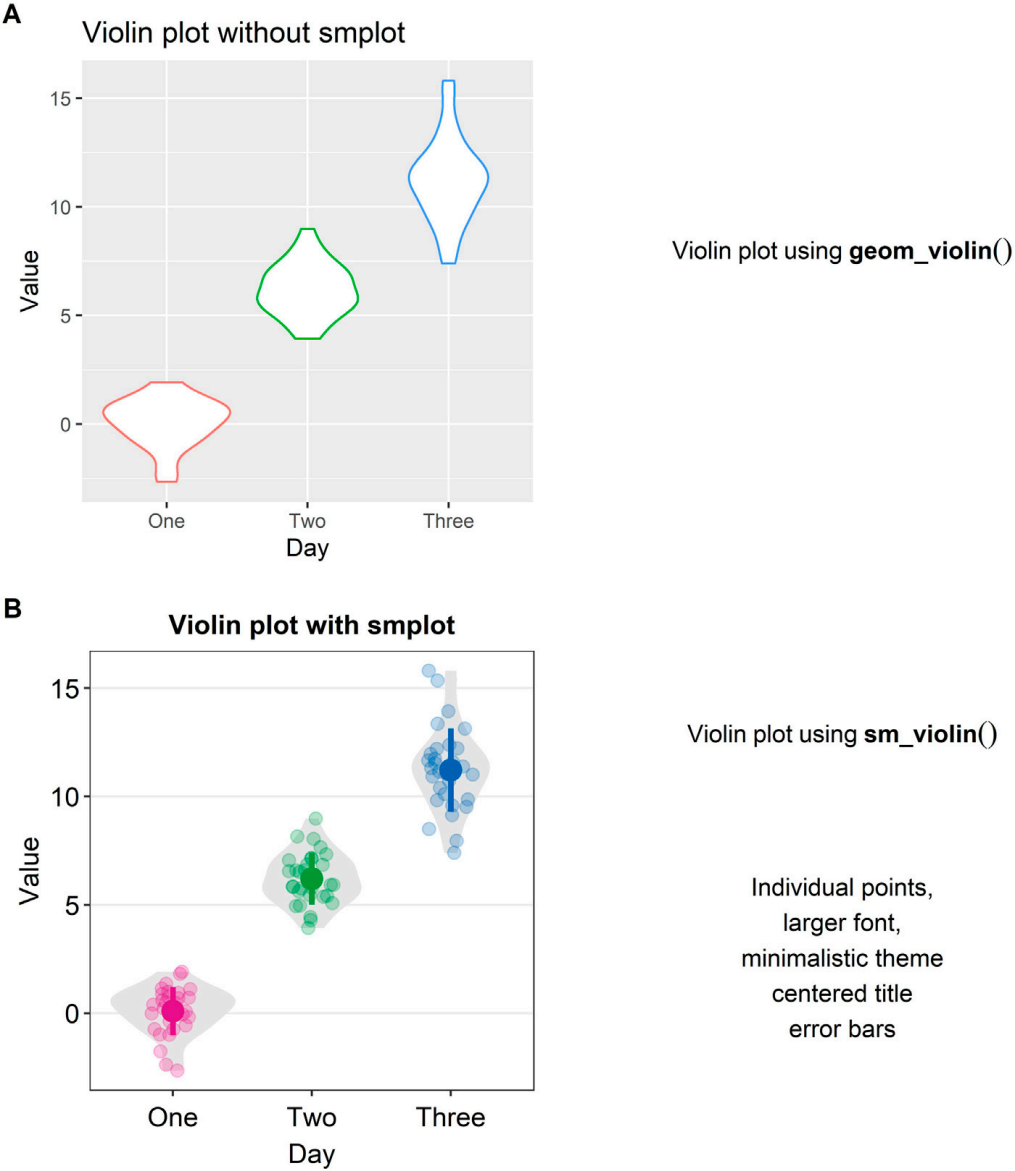
Violin plots with and without *smplot*. **(A)** A violin plot drawn with “*geom_violin()*”, which is a function of *ggplot2*. **(B)** A violin plot drawn with “*sm_violin()*”. This function automatically provides several features, such as individual data points, larger font, minimalistic theme, centered title, narrower bar width and error bars, such as standard error, standard deviation and 95% confidence interval.

Another alternative to a bar graph is a violin plot. A violin plot is sometimes preferred to a boxplot because it shows the full distribution of the data while the boxplot fails to do so. The “violin” of the violin plot represents the data distribution (see Figure 4A). The region with the largest width denotes the highest density of the data. The upper- and lowermost tips of the “violin” represent the maximum and minimum values of the data.

In Figure 4A, a violin plot using the default theme of ggplot2 (and without *smplot*) is shown. It lacks individual points and error bars, such as standard deviation. The aesthetics also need some improvement. Instead, when “*sm_violin()*” is modularly added to the plot in Figure 4A, the violin plot gets improved visually (see Figure 4B).

### Slope Chart

**FIGURE 5:**
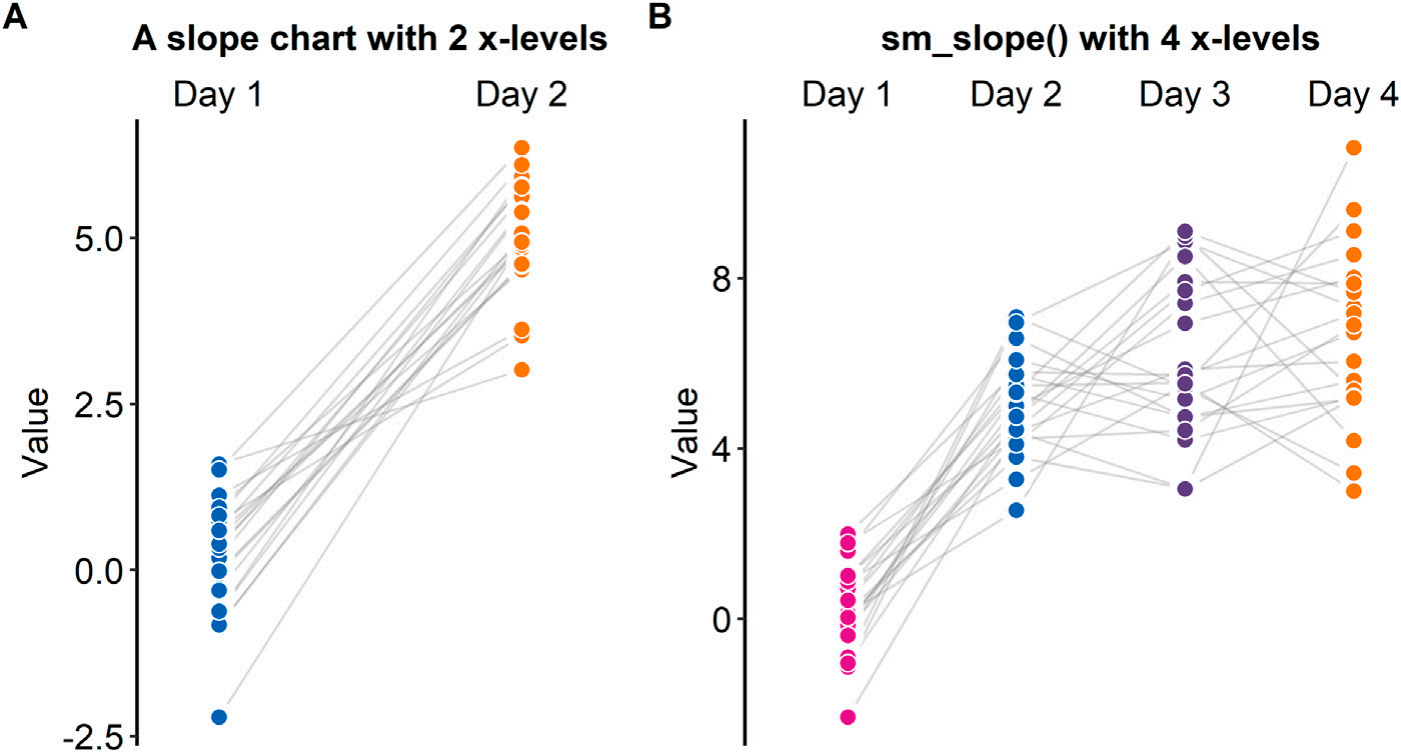
Slope charts drawn with “sm_slope *()*”, which is a function of *smplot*. **(A)** A slope chart with two discrete x-levels. **(B)** A slope chart with four discrete x-levels.

A slope chart is often used to directly compare paired data at different timepoints or instances (see Figures 5A,B). With a slope chart, one can track changes over time for each data point (i.e., before and after experimental manipulation). If one is interested in performing a statistical test that accounts for repeated measures (ex., repeated measures one-way analysis of variance), a slope chart can be a good choice for plotting data.

The *ggplot2* package does not offer a single function that plots a slope chart. To use *ggplot2*, one might need to code multiple line of code to strip away the default *ggplot2* theme and construct an appropriate slope chart, a task that can be tedious and repetitive. For this reason, “*sm_slope()*” has been created.

Figure 5A shows a slope chart that has two levels in the x-axis, whereas Figure 5B shows a slope chart that has four levels in the x-axis. “*sm_slope()*” plots these slope charts with the same command code by automatically detecting the number of discrete x-levels, provided that the loaded data has a proper data frame structure (see example: https://github.com/smin95/dataviz/blob/master/data.csv).

### Raincloud Plot = Violin Plot + Boxplot + Individual Data

A raincloud plot is a combination of a violin plot (halved), a boxplot and jittered individual data (Allen et al., 2021). Plotting a raincloud plot might be challenging for newcomers in R. Although there exists an R package (the *raincloudplots* package) that plots a raincloud plot (Allen et al., 2021), the function “*sm_raincloud()*” has been created to allow for more visual customisation.

**FIGURE 6:**
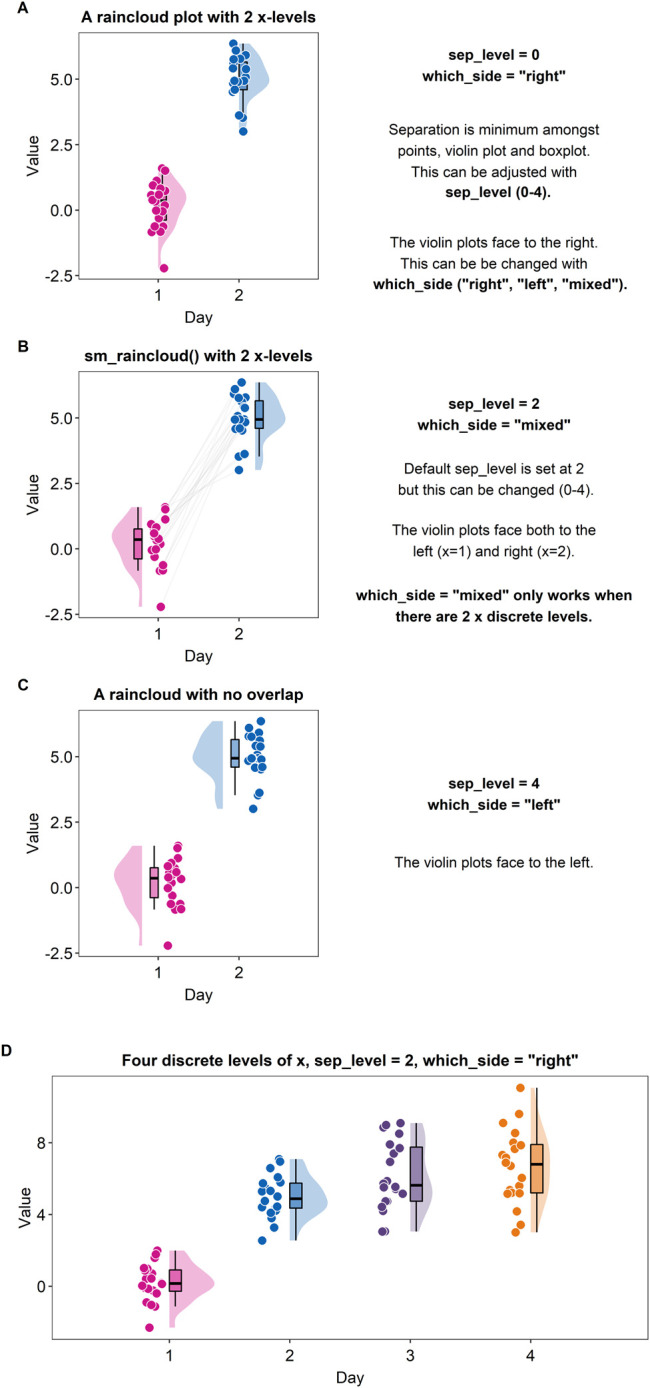
Raincloud plots drawn with “*sm_raincloud ()*”. **(A)** A raincloud plot with two discrete x-levels. In this example, the halved violin plots face to the right because the violins have been specified to face to the right, i.e., “*which_side =* “*right”*”. Also, the violin plots, boxplots and jittered individual data points all overlap among each other because the separation level has been specified to 0, i.e., “*sep_level = 0*”. **(B)** A raincloud plot with two discrete x-levels. In this example, the halved violin plots both face to the left and right, i.e., “*which_side =* “*mixed*””. This mixed configuration allows the individual points to be paired visually using the grey lines. Also, although the halved violin plots and boxplots overlap, the jittered individual data are located separately because the separation level has been specified to 2, i.e., “*sep_level = 2*”. **(C)** A raincloud plot with two discrete x-levels. In this example, the halved violin plots face to the left because the argument “*which_side =* “*left*””. Also, the halved violin plots, boxplots and the jittered individual data do not overlap. There is more separating distance than the plot in panels A and B because the separation level has been specified to 4, i.e., “*sep_level = 4*”. **(D)** A raincloud plot with four discrete x-levels.

Figure 6A shows a raincloud plot that has two discrete levels in the x-axis (Day 1 and Day 2). These levels are denoted by the distinct colors pink and blue. In this example, the jittered points, boxplot and violin plot overlap with each other because the separation level is set to 0 (“*sep_level = 0*”). “*sep_level*” is an argument for the function “*sm_raincloud()*.” The separation level ranges from 0 to 4, so one can increase the separation amongst the plots by setting “*sep_level = 2*” within the “*sm_raincloud()*” function as shown in Figure 5B. When “*sep_level = 2*,” the violin plot and the boxplot overlap each other but not the individual data points are located apart.

Another argument for “*sm_raincloud()*” is “*which_side.*” The reader may notice that the direction at which the pink violin plot is facing is to the left rather than the right (see Figure 6A). If the argument “*which_side*” is set to right, all the violin plots face to the right (see Figure 6A). However, if “*which_side = mixed*,” then the directions of the violin plots become asymmetric so that the jittered individual points at each of the two x-level are closest to one another (Figure 6B). Also, “*which_side = mixed*” is only allowed when there are two discrete levels of x-axis, and the function “*sm_raincloud()*” throws an error when the condition is not met.

In Figure 6C, separation level has been specified to 4, i.e., “*sep_level = 4.*” This allows the features of the raincloud to be separated from one another more. Also, the violin plots at each x level are facing to the left, i.e., “*which_side = left*.”

The function “*sm_raincloud()*” also plots a raincloud plot when the x-level exceeds 2 (see Figure 6D). This is a novel feature that is not included in the original R package (the *raincloudplots* package) that draws a raincloud plot. It also automatically counts the number of discrete x levels if the loaded data has a proper data frame structure (see example: https://github.com/smin95/dataviz/blob/master/data.csv).

### Case Study Using *smplot*: Test-Retest Reliability of a Novel Method

When one is interested in introducing a new measurement method, one must examine whether the new method (i.e., Method 2) shows agreeable results to those obtained from the standard method (i.e., Method 1). In this section, we present a case study where smplot might be useful. All data and codes are uploaded in Chapter 6 of the online guide (https://smin95.github.io/dataviz/).

**FIGURE 7:**
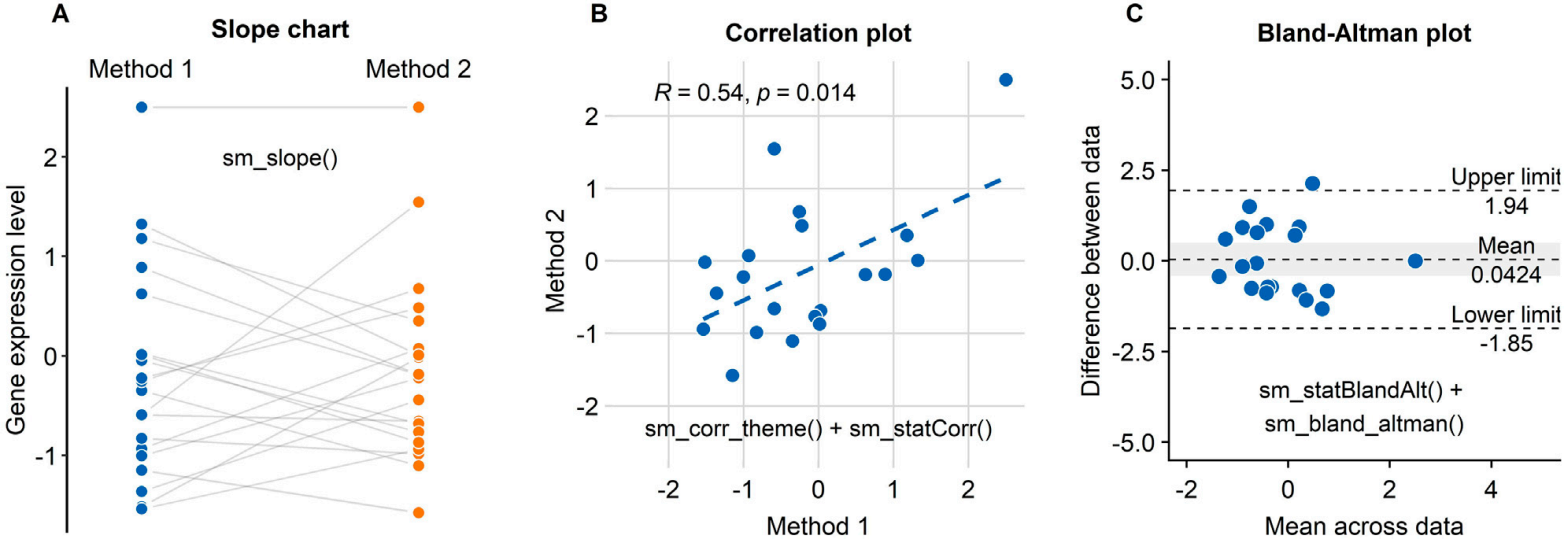
Three figures that examine the test-retest reliability of a new method/technology to measure gene expression have been created using *smplot*. **(A)** A slope chart drawn using “*sm_slope()*”. The blue points represent data from the standard method, whereas the orange points show data from the new method. Each dot represents an individual sample. The grey lines indicate the pairing of the points. If the grey lines uniformly have a positive or negative slope, then one can infer that the new method consistently show data of higher or lower values, respectively. **(B)** A correlation plot drawn using “*sm_corr_theme()*” and “*sm_statCorr()*”. Unfortunately, there is an extreme point at the top-right of the panel; it heavily skews the correlation to be robust. Without it, the correlation is much weaker, i.e., *R* = 0.24, *p* = 0.32. **(C)** A Bland-Altman plot drawn using “*sm_statBlandAlt()*” and “*sm_bland_altman()*”. The difference in data between the two methods are plotted as a function of the mean across the data from the two methods. The upper and lower dashed lines denote 95% limits of agreement; the wider the range encapsulated by the limits of agreement, the more measurement variability there is between the methods. The dashed line in the middle indicates the mean of the difference between the data from the two methods. The grey area represents 95% confidence interval of the difference in data between the methods estimated from a t-distribution. If the middle-dashed line (mean difference) does not overlap with the grey area (95% confidence interval), then the mean difference is significantly different from 0 based on one-sample t-test (*p* < 0.05), thereby indicating that the new method shows a very poor agreement with the standard method. In this panel, however, we see an overlap.

If the data across two different instances/methods are paired, one can draw a slope chart, rather than a bar plot, to see if the data between these two instances show a small variability. For instance, each dot in Figure 7A can represent an individual sample (ex. Patient 1 out of 20) from which the gene expression level is measured. In this example, if a new method is consistent with the standard method, the individual data from each specimen will have a flat grey line. As we have previously mentioned, this can be achieved by using the “*sm_slope()*” function.

Another popular method to demonstrate that a new technique is reliable is to compute whether there is a high correlation coefficient (see Figure 7B; Pearson’s correlation test is provided in this example). Unfortunately, a high correlation does not indicate a good replicability. In Figure 7B, we see that the correlation is robust (*R* = 0.64, *p* = 0.014). However, the correlation seems to be heavily dependent on one single point in the top-right corner of Figure 7B (Method 1 = 2.5, Method 2 = 2.5). If the correlation coefficient is computed without the extreme point, it might be more representative of whether the new method is truly correlated with the standard method. In this example, the correlation without the extreme point turns out to be weak, *R* = 0.24, *p* = 0.32. If the reader encounters a similar situation to this case study, we suggest that the reader compute the correlation with and without the outlier, and then determine which of the two correlation coefficients is more representative.

An appropriate approach to report test-retest variability is to show a Bland-Altman plot (see Figure 7C), which is also known as a MA plot (M = minus, A = average) in the field of genomics (Bland and Altman, 1986; Giavarina, 2015). The y-axis of the Bland-Altman is the difference between data from the two methods, whereas the x-axis denotes the mean of the data from the two methods. This plot aims to describe agreements between data from two instances. Bland and Altman have stated that 95% of the scatter points in a Bland-Altman plot should reside within the limits of agreement (dashed line in Figure 6C), which represent ±1.96 standard deviations from the mean difference between data from two sessions (Bland and Altman, 1986). Whether the mean difference between two instances is too large or not can be determined by calculating the mean difference of all paired individual data. If the mean difference of the data is not significantly different from 0 (i.e., one-sample t-test), then it is acceptable to surmise from the given data that there is a good agreement between the two methods. This is also the case in Figure 7C. A Bland-Altman plot can be drawn using these two functions “*sm_statBlandAlt()*” and “*sm_bland_altman()*.”

### Who Is smplot for?

The *smplot* package is for those who is interested in plotting elegant graphs with minimal codes in a modular fashion. It aims to simplify the process of data visualization for incoming research trainees in fields such as biomedical sciences. That being said, it is not necessary to produce high-quality graphs. We have encountered numerous medical students who use multiple software environments to create and polish figures, a process that is often laborious and tedious. For instance, if students have already created a figure and decide to collect additional data, they will find themselves to change their figures across multiple software platforms, such as Matlab and Adobe Illustrator. We hope to have convinced the reader that *smplot* can be used to create a polished, satisfying figure within one software environment with minimal coding.

If the reader is interested in learning more about R, please consider reading *R for Data Science* by Hadley Wickham (Wickham and Grolemund, 2016). If the reader is interested in developing her own color palette, please visit the online guide of Seaborn (https://seaborn.pydata.org/tutorial/color_palettes.html), which is a data visualization library in Python (Waskom, 2021). If the reader is interested in learning important practices of data visualization, please consider reading *Fundamentals of Data Visualization* by Claus Wilke (Wilke, 2019b); he is the author of the *cowplot* package (Wilke, 2019a).

### Contributions of the Package

The smplot package provides numerous functions that quicken the process of data visualization. Most functions are wrapper functions around ggplot2 that aim to change the default of the aesthetics. We also provide new functions, such as “*sm_bland_altman()*” and “*sm_raincloud()*” that do more than changing the default theme of ggplot2. “*sm_bland_altman()*” plots a Bland-Altman plot (along with the mean, upper and lower limits) and a grey shaded region that represents the 95% confidence interval; these labels are all necessary but the R package (ex. the *BlandAltmanLeh* package) for Bland-Altman plots do not necessarily plot all of these features by default and do not use the ggplot2 interface. Moreover, “*sm_raincloud()*” draws a raincloud plot that is more customisable than the original package that draws a raincloud plot (the *raincloudplots* package). smplot does not impose the limits of the number of discrete x-levels unlike the original package (the *raincloudplots* package). For example, the *raincloudplots* package is not capable of plotting Figure 6D because the graph requires 4 discrete x-levels. In addition, the configuration of the violin plots in the raincloud plot as well as the aesthetics can also be more customised than before. Lastly, unlike the *raincloudplots* package, the data structure can have the same format as the one required for ggplot2; this consistency of the data structure between raincloud plots and other ggplot2 figures can allow the user to draw multiple graphs without modifying the data structure.

The *ggpubr* package is a well-known R package for data visualization. However, many plotting functions of the *ggpubr* package are one-liner, rather than modular, functions that plot a complete graph. For this reason, there are numerous stored defaults that might not be accessible for the user to modify. If a modular function is added to a plot that is created with *ggpubr* to change default aesthetics of *ggpubr*, warnings may appear. For this reason, the *smplot* package provides functions that can be added modularly (ex. “*sm_hgrid*” and “*sm_statCorr*”) to the given plot built with ggplot2 or be added to (“*sm_bland_altman*” and “*sm_raincloud*”) by other modular functions.

The smplot package provides multiple themes with an interesting feature. First, as is the case of the themes of the *cowplot* package, they can be added in a modular fashion to a given ggplot2 plot (ex. base plot “*+ sm_hgrid()*”). Also, the theme functions of smplot provide a separate argument for the border and the legend (ex. “*sm_hgrid(legends = FALSE, borders = TRUE*).” If “*legends = FALSE*,” the legend will be hidden; if “*borders = TRUE,*” there will be a border around the panel. When these settings are flipped (“*legends = TRUE*” and “*borders = TRUE*”), the relative proportion of the figure as well as the perceived size of the text have been set to appear the same. These features have been added for convenience because the user is otherwise forced to use “*theme()*,” which can be tedious and confusing to use. The themes provided by the *cowplot* package do not offer these features.

## Data Availability

The *smplot* R package is free and open source. All sample data and codes of the figures can be accessed in the online guide (https://smin95.github.io/dataviz/). The source codes of smplot are available in Github (https://github.com/smin95/smplot). *smplot* requires *ggplot2* (Wickham, 2016) and *cowplot* (Wilke, 2019a) packages, both of which are automatically downloaded when *smplot* is installed via RStudio. Please cite this article when smplot is used.
