# Supplementary material for: smplot: An R Package for Easy and Elegant Data Visualization
Source: Front Genet. 2021 Dec 15;12:802894. doi: 10.3389/fgene.2021.802894 (PMC8714909; doi:10.3389/fgene.2021.802894)
Supplement: Supplementary file 1 [file DataSheet1.PDF]

Recreating the Manuscript Figures

This supplmentary material is Chapter 6 of the online guide entitled **Data Visualization in R Using smplot** (https://smin95.github.io/dataviz/).

```
library(tidyverse) # it has ggplot2 package
```

```
## -- Attaching packages --------------------------------------- tidyverse 1.3.1 --

## v ggplot2 3.3.3     v purrr   0.3.4
## v tibble  3.1.1     v dplyr   1.0.5
## v tidyr   1.1.3     v stringr 1.4.0
## v readr   1.4.0     v forcats 0.5.1

## -- Conflicts ------------------------------------------ tidyverse_conflicts() --
## x dplyr::filter() masks stats::filter()
## x dplyr::lag()    masks stats::lag()
```

```
library(cowplot) # it allows you to save figures in .png file
library(smplot)
```

```
## Updated tutorial for smplot: smin95.github.io/dataviz/
```

Figure 1 - Correlation plot

```
corr1 <- ggplot(data = mtcars, mapping = aes(x = drat, y = mpg)) +
  geom_point(shape = 21, fill = sm_color('green'), color = 'white',
             size = 3) + # ggplot2 default
  ggtitle('Correlation plot without smplot')

text1 <- ggplot(data = mtcars, mapping = aes(x = drat, y = mpg)) +
  geom_point(color = 'white', fill = 'white') + theme_nothing() +
  annotate('text',
           label = 'Base plot only using \nthe defaults of ggplot2',
           x = 3.9, y = 25)

corr2 <- ggplot(data = mtcars, mapping = aes(x = drat, y = mpg)) +
  geom_point(shape = 21, fill = sm_color('green'), color = 'white',
             size = 3) +
  sm_corr_theme() + # smplot correlation theme
  ggtitle('Correlation plot with smplot')


text2 <- ggplot(data = mtcars, mapping = aes(x = drat, y = mpg)) +
  geom_point(color = 'white', fill = 'white') + theme_nothing() +
  annotate('text', label = 'Base plot + sm_corr_theme()',
           x = 3.9, y = 29, fontface = 2) +
  annotate('text', x = 3.9, y = 23, parse = TRUE,
           label = 'bold(sm_corr_theme())~\nprovides~\na~theme~with') +
  annotate('text', x = 3.9, y = 20,
           label = 'minimalistic background\nlarger font\ncentered title')

corr3 <- ggplot(data = mtcars, mapping = aes(x = drat, y = mpg)) +
  geom_point(shape = 21, fill = sm_color('green'), color = 'white',
             size = 3) +
  sm_corr_theme() + # smplot corr theme + p and r values
```

```r
  sm_statCorr(color = sm_color('green'), corr_method = 'spearman') +
  ggtitle('Statistics computed with smplot')

text3 <- ggplot(data = mtcars, mapping = aes(x = drat, y = mpg)) +
  geom_point(color = 'white', fill = 'white') + theme_nothing() +
  annotate('text', label = 'Base plot + sm_corr_theme() +\nsm_statCorr()',
           x = 3.9, y = 30,
           fontface = 2) +
  annotate('text', x = 3.9, y = 24, parse = TRUE,
           label = 'bold(sm_statCorr())~from~smplot~computes') +
  annotate('text', x = 3.9, y = 22.6,
           label = 'R and p values from a correlation test.') +
  annotate('text', x = 3.9, y = 18,
           label = '\nIt also prints the best-fit \nlinear regression line \nbased on the R value.')

corr_all <- plot_grid(corr1, text1, corr2, text2,
                      corr3, text3,
                 labels = c("A", "", "B", "", "C", ""),
                 label_size = 12,
                 ncol = 2, nrow = 3,
                 scale = 0.95)
```

```
## `geom_smooth()` using formula 'y ~ x'
```

```r
print(corr_all)
```

A

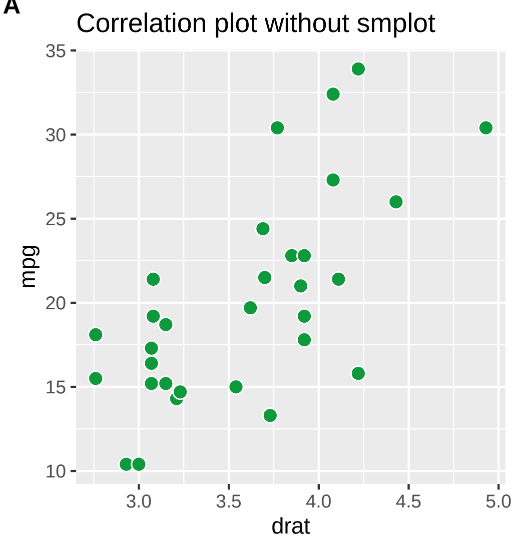

Base plot only using
the defaults of ggplot2

B

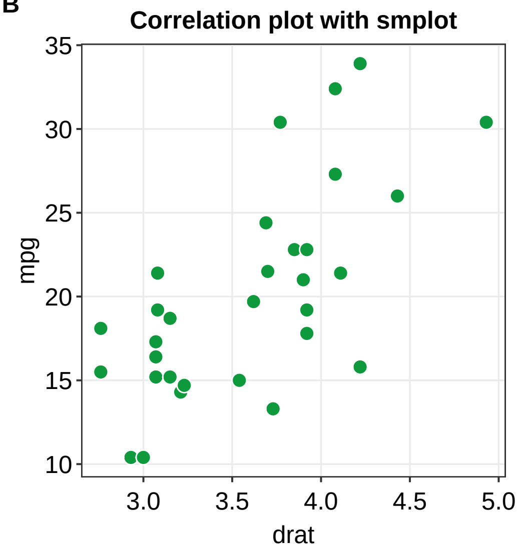

Base plot + sm_corr_theme()

sm_corr_theme() provides a theme with
minimalistic background
larger font
centered title

C

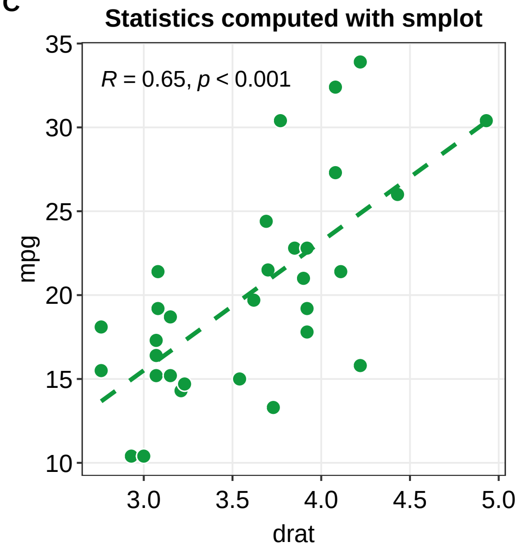

Base plot + sm_corr_theme() +
sm_statCorr()

sm_statCorr() from smplot computes
R and p values from a correlation test.

It also prints the best−fit
linear regression line
based on the R value.

```
save_plot("corr_plots.png",
          corr_all, ncol = 2, nrow = 3, base_asp = .95,
          dpi = 600)
```

Figure 2 - Bar plot

```
set.seed(1) # generate random data
day1 = rnorm(20,0,1)
day2 = rnorm(20,5,1)
Subject <- rep(paste0('S',seq(1:20)), 2)
Data <- data.frame(Value = matrix(c(day1,day2),ncol=1))
Day <- rep(c('Day 1', 'Day 2'), each = length(day1))
df <- cbind(Subject, Data, Day)

bar1 <- ggplot(data = df, mapping = aes(x = Day, y = Value, fill = Day)) +
  stat_summary(fun = mean, geom = 'bar') +
  theme(legend.position = 'none') +
  ggtitle('Bar plot without smplot')

text1 <- ggplot(data = df, mapping = aes(x = Day, y = Value)) +
  geom_point(color = 'white', fill = 'white') + theme_nothing() +
  annotate('text',
           label = 'Bar~plot~using~bold(stat_summary())~from~ggplot2',
           x = 1.5, y = 3, parse = TRUE)

bar2 <- ggplot(data = df, mapping = aes(x = Day, y = Value, fill = Day)) +
  sm_bar(shape = 21, color = 'white', bar_fill_color = 'gray80') +
  scale_fill_manual(values = sm_color('blue','orange')) +
  ggtitle('Bar plot using smplot')

text2 <- ggplot(data = df, mapping = aes(x = Day, y = Value)) +
  geom_point(color = 'white', fill = 'white') + theme_nothing() +
  annotate('text', label = 'Bar~plot~using~bold(sm_bar())',
           x = 1.5, y = 4.5, parse = TRUE) +
  annotate('text',
           label = 'Individual points,\nlarger font,\nminimalistic theme\ncentered title\nnarrower bar [illegible]
           x = 1.5, y = 1.5)
# \nnarrower bar width\nerror bar'
bar_all <- plot_grid(bar1, text1, bar2, text2,
                        labels = c("A", "", "B", ""),
                        label_size = 12,
                        ncol = 2, nrow = 2,
                        scale = 0.95)

print(bar_all)
```

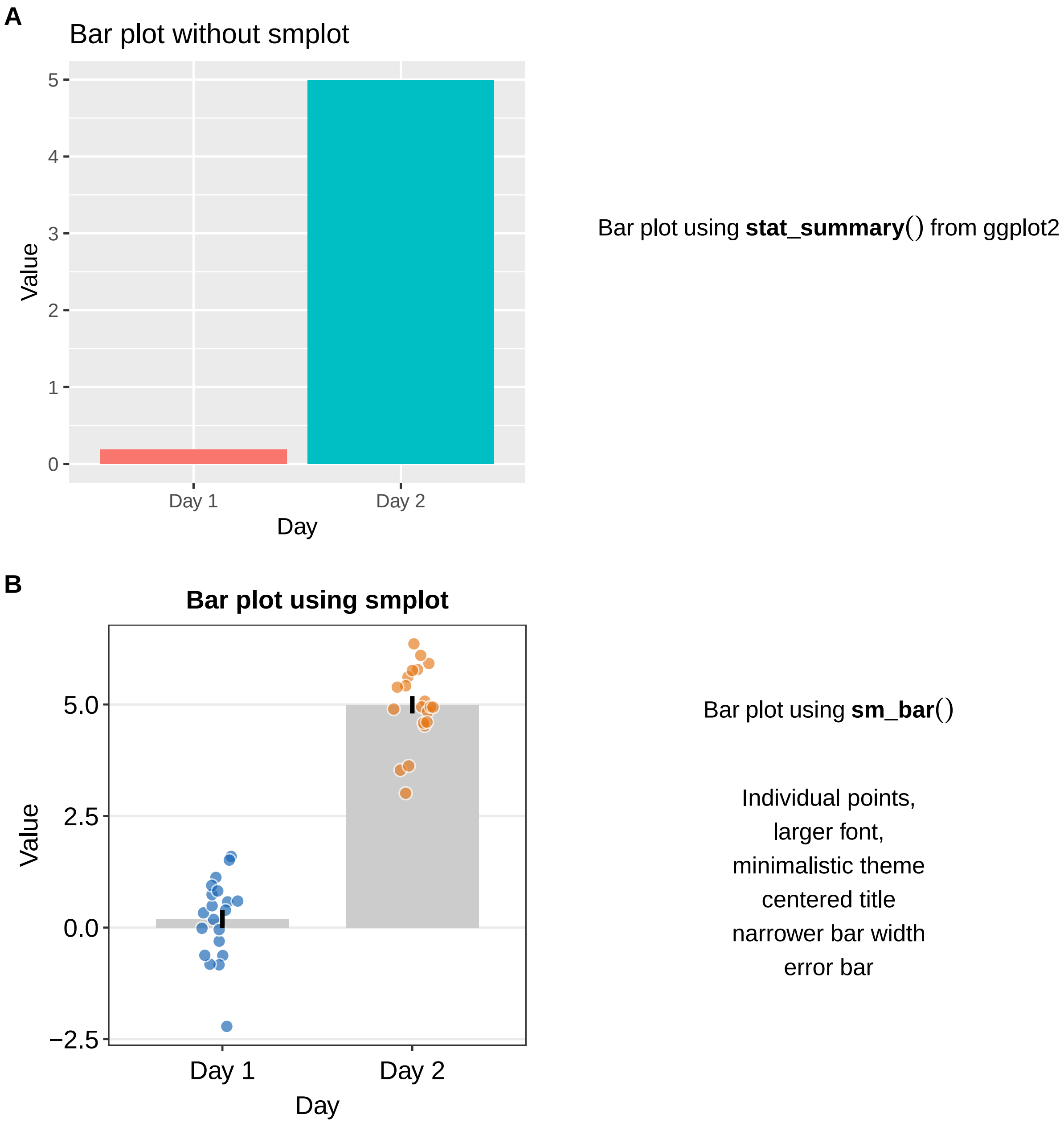


```
save_plot("bars.png",
          bar_all, ncol = 2, nrow = 2, base_asp = .95,
          dpi = 600)
```

Figure 3 - Boxplot

```
df <- read_csv('https://raw.githubusercontent.com/smin95/dataviz/master/data.csv')
```

```
##
## -- Column specification --------------------------------------------------------
## cols(
##   Subject = col_character(),
##   Value = col_double(),
```

```
##   Day = col_character()
## )
```

```r
df$Day <- factor(df$Day, levels = c('One','Two','Three'))

boxplot1 <- ggplot(data = df, mapping = aes(x = Day, y = Value, color = Day)) +
  geom_boxplot() +
  theme(legend.position = 'none') +
  ggtitle('Boxplot without smplot')

text1 <- ggplot(data = df, mapping = aes(x = Day, y = Value)) +
  geom_point(color = 'white', fill = 'white') + theme_nothing() +
  annotate('text',
           label = 'Boxplot~using~bold(geom_boxplot())\n~from~ggplot2',
           x = 2, y = 8, parse = TRUE)

boxplot2 <- ggplot(data = df, mapping = aes(x = Day, y = Value, color = Day)) +
  sm_boxplot(shape = 16, alpha = 0.4) +
  scale_color_manual(values = sm_palette(3)) +
  ggtitle('Boxplot with smplot') # smplot's default

text2 <- ggplot(data = df, mapping = aes(x = Day, y = Value)) +
  geom_point(color = 'white', fill = 'white') + theme_nothing() +
  annotate('text',
           label = 'Boxplot~using~bold(sm_boxplot())',
           x = 2, y = 11, parse = TRUE) +
  annotate('text',
           label = 'Individual points,\nlarger font,\nminimalistic theme\ncentered title',
           x = 2, y = 5)

boxplot_all <- plot_grid(boxplot1, text1, boxplot2, text2,
                      labels = c("A", "", "B", ""),
                      label_size = 12,
                      ncol = 2, nrow = 2,
                      scale = 0.95)

print(boxplot_all)
```

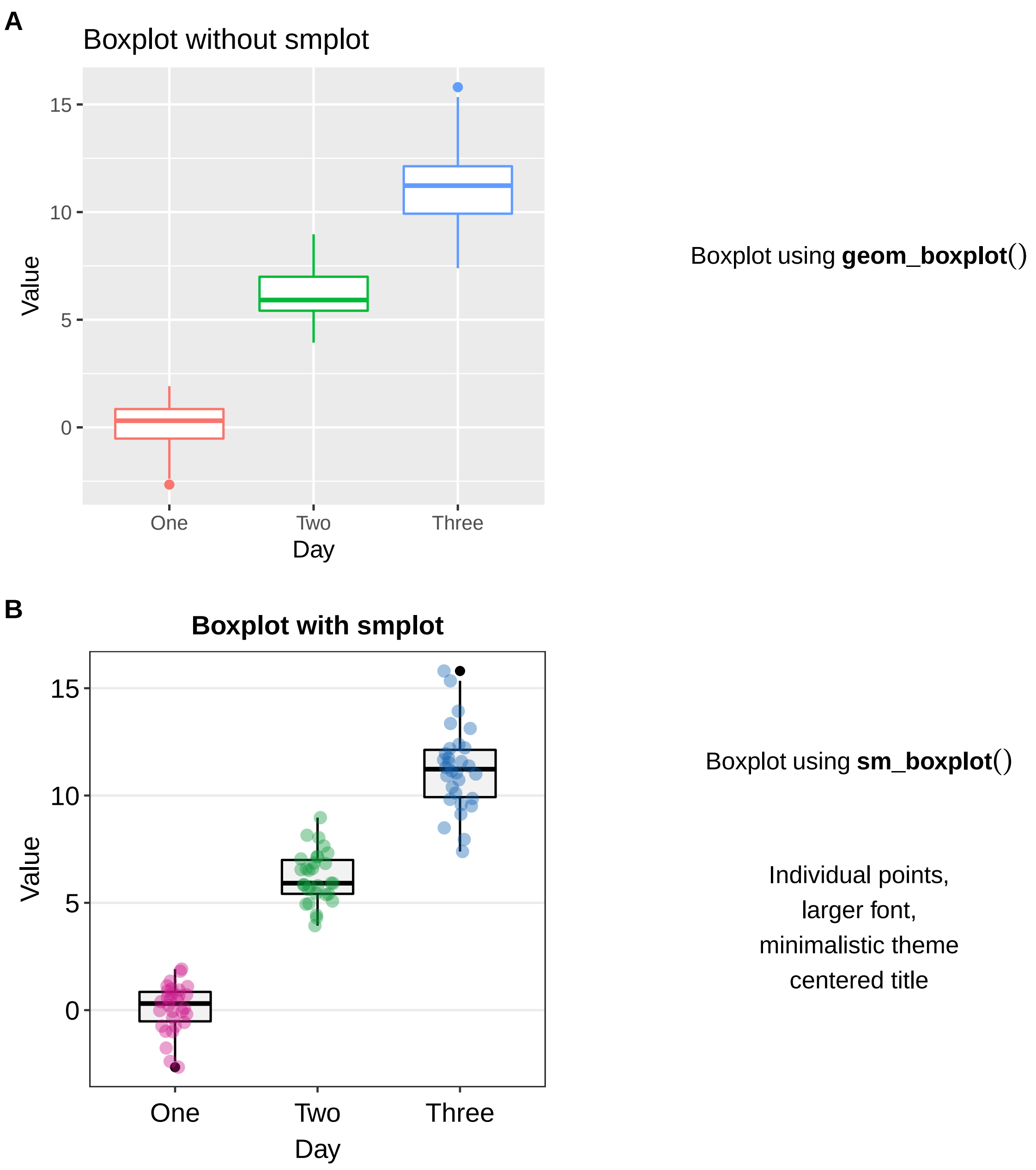


```
save_plot("boxplots.png",
          boxplot_all, ncol = 2, nrow = 2, base_asp = .95,
          dpi = 600)
```

Figure 4 - Violin plot

```
violin1 <- ggplot(data = df, mapping = aes(x = Day, y = Value, color = Day)) +
  geom_violin() +
  theme(legend.position = 'none') +
  ggtitle('Violin plot without smplot') ## ggplot2 default

text1 <- ggplot(data = df, mapping = aes(x = Day, y = Value)) +
  geom_point(color = 'white', fill = 'white') + theme_nothing() +
```

```
  annotate('text',
           label = 'Violin~plot~using~bold(geom_violin())\n~from~ggplot2',
           x = 2, y = 8, parse = TRUE)

violin2 <- ggplot(data = df, mapping = aes(x = Day, y = Value, color = Day)) +
  sm_violin() +
  scale_color_manual(values = sm_palette(3)) +
  ggtitle('Violin plot with smplot') # smplot default

text2 <- ggplot(data = df, mapping = aes(x = Day, y = Value)) +
  geom_point(color = 'white', fill = 'white') + theme_nothing() +
  annotate('text',
           label = 'Violin~plot~using~bold(sm_violin())\nfrom~smplot',
           x = 2, y = 12, parse = TRUE) +
  annotate('text',
           label = 'Individual points,\nlarger font,\nminimalistic theme\ncentered title\nerror bars',
           x = 2, y = 4)

violin_all <- plot_grid(violin1, text1, violin2, text2,
                         labels = c("A", "", "B", ""),
                         label_size = 12,
                         ncol = 2, nrow = 2,
                         scale = 0.95)

print(violin_all)
```

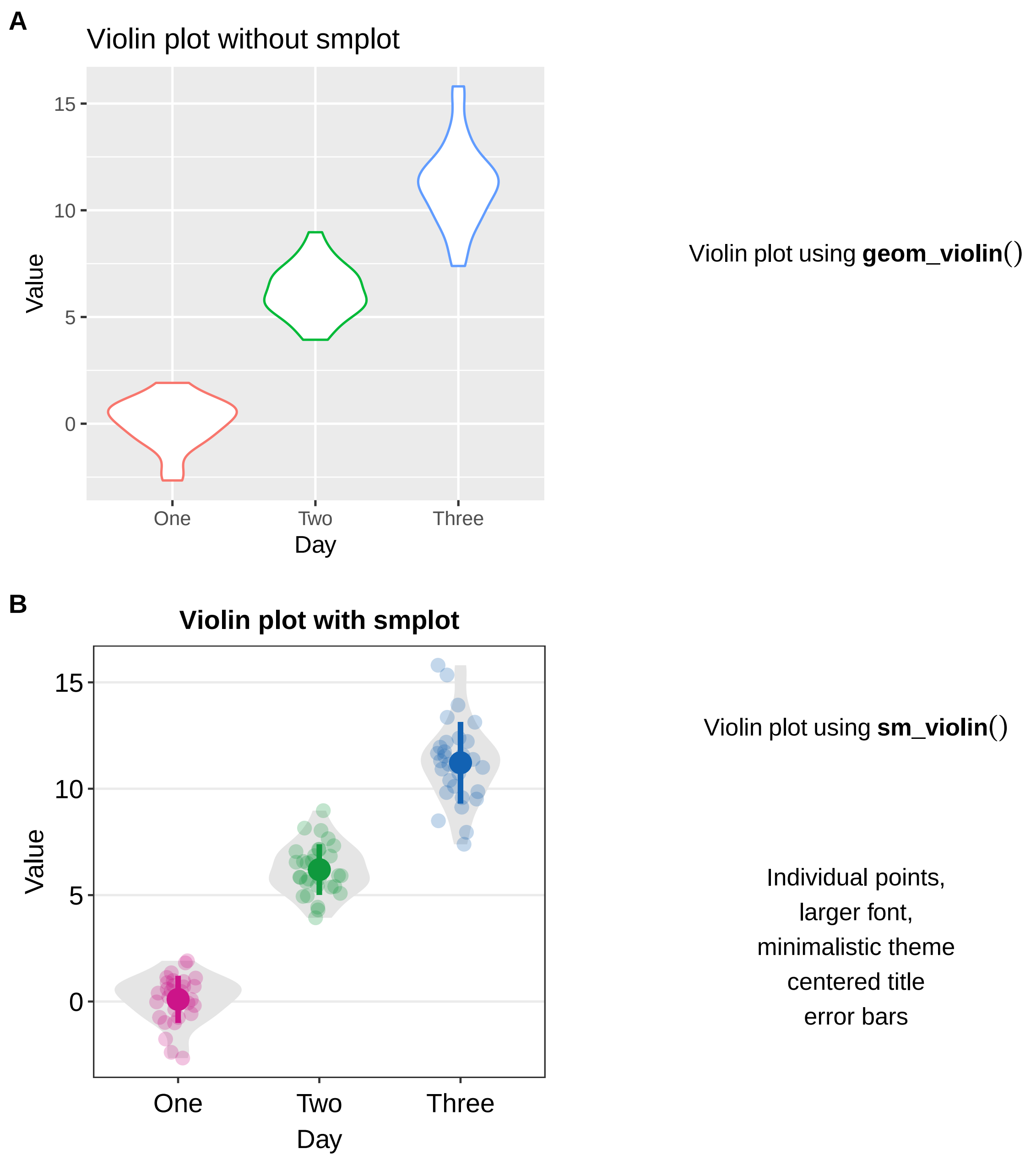


```
save_plot("violins.png",
          violin_all, ncol = 2, nrow = 2, base_asp = .95,
          dpi = 600)
```

Figure 5 - Slope chart

```
set.seed(1) # generate random data
day1 = rnorm(20,0,1)
day2 = rnorm(20,5,1)
Subject <- rep(paste0('S',seq(1:20)), 2)
Data <- data.frame(Value = matrix(c(day1,day2),ncol=1))
Day <- rep(c('Day 1', 'Day 2'), each = length(day1))
df1 <- cbind(Subject, Data, Day)
```

```r
ggplot(data = df1, mapping = aes(x = Day, y = Value,
                                 group = Subject, fill = Day)) +
  sm_slope(labels = c('Day 1', 'Day 2'),
           shape = 21, color = 'white', line_alpha = 0.3) +
  scale_fill_manual(values = sm_color('blue','orange')) +
  ggtitle('A slope chart with 2 x-levels') +
  theme(plot.title = element_text(face="bold")) -> slope_n2

set.seed(2) # generate random data
day1 = rnorm(20,0,1)
day2 = rnorm(20,5,1)
day3 = rnorm(20,6,1.5)
day4 = rnorm(20,7,2)
Subject <- rep(paste0('S',seq(1:20)), 4)
Data <- data.frame(Value = matrix(c(day1,day2,day3,day4),ncol=1))
Day <- rep(c('Day 1', 'Day 2', 'Day 3', 'Day 4'), each = length(day1))
df2 <- cbind(Subject, Data, Day)

ggplot(data = df2, mapping = aes(x = Day, y = Value,
                                 group = Subject, fill = Day)) +
  sm_slope(labels = c('Day 1', 'Day 2', 'Day 3', 'Day 4'),
           shape = 21, color = 'white', line_alpha = 0.3) +
  scale_fill_manual(values = sm_palette(4)) +
  ggtitle('sm_slope() with 4 x-levels') +
  theme(plot.title = element_text(face="bold")) -> slope_n4

slopes <- plot_grid(slope_n2, slope_n4,
          labels = c("A", "B"), rel_widths = c(1.5,2),
          label_size = 12,
          ncol = 2, nrow = 1,
          scale = 0.95)

print(slopes)
```

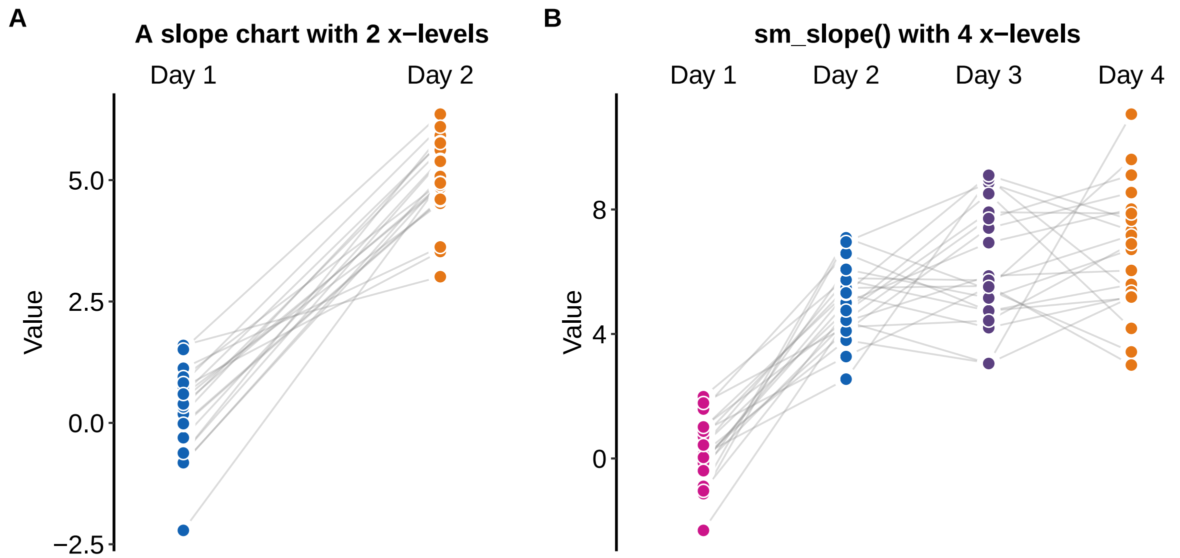

```
save_plot("slopes.png",
          slopes, ncol = 2, nrow = 1, base_asp = .95,
          dpi = 600)
```

Figure 6 - Raincloud plot

```
raincloud1 <- sm_raincloud(data = df1, x = Day, y = Value,
             boxplot_alpha = 0.5, color = 'white', shape = 21,
             sep_level = 0, line_alpha = 0.2) +
  scale_x_continuous(limits = c(0.15,2.85),
                     labels = c('1', '2'),
                     breaks = c(1,2)) +
  scale_color_manual(values = rep('transparent',2)) +
  scale_fill_manual(values = sm_palette(2)) +
  ggtitle('A raincloud plot with 2 x-levels') +
  xlab('Day') +
  theme(plot.title = element_text(face="bold"))

text1 <- ggplot(data = df1, mapping = aes(x = Day, y = Value)) +
  geom_point(color = 'white', fill = 'white') + theme_nothing() +
  annotate('text', label = 'sep_level = 0\nwhich_side = "right"',
           x = 1.5, y = 5, fontface = 2) +
  annotate('text', x = 1.5, y = 2.5,
           label = 'Separation is minimum amongst\npoints, violin plot and boxplot.\nThis can be adjust
  #\nThis can be adjusted with')
  annotate('text', x = 1.5, y = 1.6,
           label = '\nsep_level (0-4).', fontface = 2) +
  annotate('text', x = 1.5, y = -0.5,
           label = 'The violin plots face to the right.\nThis can be be changed with') +
  annotate('text', x = 1.5, y = -1.1,
           label = '\nwhich_side ("right", "left", "mixed").',
           fontface = 2)


raincloud2 <- sm_raincloud(data = df1, x = Day, y = Value, group = Subject,
             boxplot_alpha = 0.5, color = 'white', shape = 21,
             sep_level = 2, line_alpha = 0.2, which_side = 'mixed') +
  scale_x_continuous(limits = c(0.15,2.85),
                     labels = c('1', '2'),
                     breaks = c(1,2)) +
  scale_color_manual(values = rep('transparent',2)) +
  scale_fill_manual(values = sm_palette(2)) +
  ggtitle('sm_raincloud() with 2 x-levels') +
  xlab('Day') +
  theme(plot.title = element_text(face="bold"))

text2 <- ggplot(data = df1, mapping = aes(x = Day, y = Value)) +
  geom_point(color = 'white', fill = 'white') + theme_nothing() +
  annotate('text', label = 'sep_level = 2\nwhich_side = "mixed"',
           x = 1.5, y = 4.7, fontface = 2) +
  annotate('text', x = 1.5, y = 2.8,
           label = 'Default sep_level is set at 2\n but this can be changed (0-4).') +
  annotate('text', x = 1.5, y = 1,
```

```
          label = 'The violin plots face both to the\nleft (x=1) and right (x=2).') +
  annotate('text', x = 1.5, y = -0.6,
          label = '\nwhich_side = "mixed" only works when\nthere are 2 x discrete levels.',
          fontface = 2)

raincloud3 <- sm_raincloud(data = df1, x = Day, y = Value,
             boxplot_alpha = 0.5, color = 'white', shape = 21,
             sep_level = 4, line_alpha = 0.2, which_side = 'left') +
  scale_x_continuous(limits = c(0.15,2.85),
                     labels = c('1', '2'),
                     breaks = c(1,2)) +
  scale_color_manual(values = rep('transparent',2)) +
  scale_fill_manual(values = sm_palette(2)) +
  ggtitle('A raincloud with no overlap') +
  xlab('Day') +
  theme(plot.title = element_text(face="bold"))

text3 <- ggplot(data = df1, mapping = aes(x = Day, y = Value)) +
  geom_point(color = 'white', fill = 'white') + theme_nothing() +
  annotate('text', label = 'sep_level = 4\nwhich_side = "left"',
          x = 1.5, y = 3.5, fontface = 2) +
  annotate('text', x = 1.5, y = 1.5,
           label = 'The violin plots face to the left.')

raincloud_2x_all <- plot_grid(raincloud1, text1,
                              raincloud2, text2,
                              raincloud3, text3,
                     labels = c("A", "", "B", "", "C", ""),
                     label_size = 12,
                     ncol = 2, nrow = 3,
                     scale = 0.95)

raincloud4 <- sm_raincloud(data = df2, x = Day, y = Value,
             boxplot_alpha = 0.5, color = 'white', shape = 21,
             sep_level = 2, line_alpha = 0.2) +
  scale_x_continuous(limits = c(0.25,4.75),
                     labels = c('1', '2', '3', '4'),
                     breaks = c(1,2,3,4)) +
  scale_color_manual(values = rep('transparent',4)) +
  scale_fill_manual(values = sm_palette(4)) +
  ggtitle('Four discrete levels of x, sep_level = 2, which_side = "right"') +
  xlab('Day') +
  theme(plot.title = element_text(face="bold"))

raincloud_all <- plot_grid(raincloud_2x_all,
                           raincloud4,
                           labels = c("","D"),
                           label_size = 12,
                           ncol = 1, nrow = 2,
                           scale = 0.95,
                           rel_heights = c(7.5,2.5))

print(raincloud_all)
```

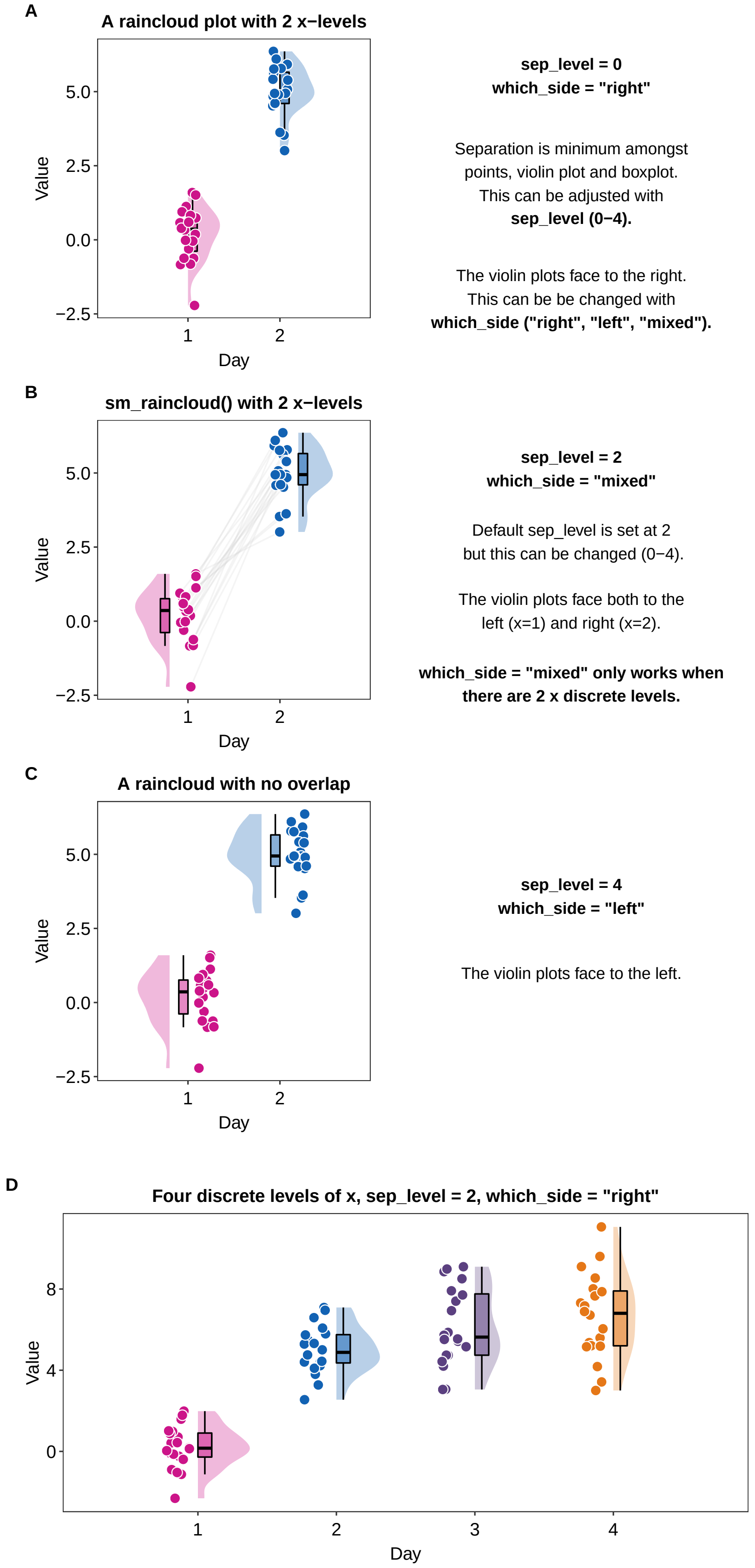
A
A raincloud plot with 2 x−levels
sep_level = 0
which_side = "right"
Separation is minimum amongst points, violin plot and boxplot. This can be adjusted with sep_level (0−4).
The violin plots face to the right. This can be be changed with which_side ("right", "left", "mixed").
B
sm_raincloud() with 2 x−levels
sep_level = 2
which_side = "mixed"
Default sep_level is set at 2 but this can be changed (0−4).
The violin plots face both to the left (x=1) and right (x=2).
which_side = "mixed" only works when there are 2 x discrete levels.
C
A raincloud with no overlap
sep_level = 4
which_side = "left"
The violin plots face to the left.
D
Four discrete levels of x, sep_level = 2, which_side = "right"
Value
Day

```
save_plot("rainclouds.png",
          raincloud_all, ncol = 2, nrow = 4, base_asp = .95,
          dpi = 600)
```

Figure 7 - Case study

```
set.seed(11) # generate random data
method1 = c(rnorm(19,0,1),2.5)
method2 = c(rnorm(19,0,1),2.5)
Subject <- rep(paste0('S',seq(1:20)), 2)
Data <- data.frame(Value = matrix(c(method1,method2),ncol=1))
Method <- rep(c('Method 1', 'Method 2'), each = length(method1))
df <- cbind(Subject, Data, Method)

# slope chart
ggplot(data = df, mapping = aes(x = Method, y = Value,
                                 group = Subject, fill = Method)) +
  sm_slope(labels = c('Method 1', 'Method 2'),
           shape = 21, color = 'white', line_alpha = 0.3) +
  scale_fill_manual(values = sm_color('blue','orange')) +
  ggtitle('Slope chart') +
  theme(plot.title = element_text(face="bold")) +
  ylab('Gene expression level') +
  annotate('text', x = 1.5, y = 2, label = 'sm_slope()') -> slope

# correlation plot

df3 <- data.frame(first = method1, second = method2)

corr_plot <- ggplot(data = df3, mapping = aes(x = first, y = second)) +
  geom_point(shape = 21, fill = sm_color('blue'), color = 'white',
             size = 3) + sm_corr_theme(borders = FALSE) +
  scale_y_continuous(limits = c(-2.5,2.5)) +
  scale_x_continuous(limits = c(-2.5,2.5)) +
  sm_statCorr(color = sm_color('blue'), corr_method = 'pearson',
              label_x = -2.2, label_y = 2.3) +
  ggtitle('Correlation plot') +
  xlab('Method 1') + ylab('Method 2') +
  annotate('text', x = 0, y = -2.3, label = 'sm_corr_theme() + sm_statCorr()')

# BA plot
res <- sm_statBlandAlt(df3$first,df3$second)

ba_plot <- sm_bland_altman(df3$first, df3$second, shape = 21,
                color = 'white',
                fill = sm_color('blue')) +
  scale_y_continuous(limits = c(-5,5)) +
  scale_x_continuous(limits = c(-2,5)) +
  annotate('text', label = 'Mean', x = 4.3, y = res$mean_diff + 0.4) +
  annotate('text', label = signif(res$mean_diff,3), x = 4.3, y = res$mean_diff - 0.4) +
  annotate('text', label = 'Upper limit', x = 4.3, y = res$upper_limit + 0.4) +
  annotate('text', label = signif(res$upper_limit,3), x = 4.3, y = res$upper_limit - 0.4) +
  annotate('text', label = 'Lower limit', x = 4.3, y = res$lower_limit + 0.4) +
```

```
  annotate('text', label = signif(res$lower_limit,3), x = 4.3, y = res$lower_limit - 0.4) +
  ggtitle('Bland-Altman plot') +
  annotate('text', x = 1.5, y = -4, label = 'sm_statBlandAlt() + \nsm_bland_altman()')

case_study <- plot_grid(slope, corr_plot, ba_plot,
                      labels = c("A", "B", "C"),
                      hjust = -4, # horizontal position of ABC labels
                      label_size = 12,
                      ncol = 3, nrow = 1,
                      scale = 0.95)
```

```
## `geom_smooth()` using formula 'y ~ x'
```

```
print(case_study)
```

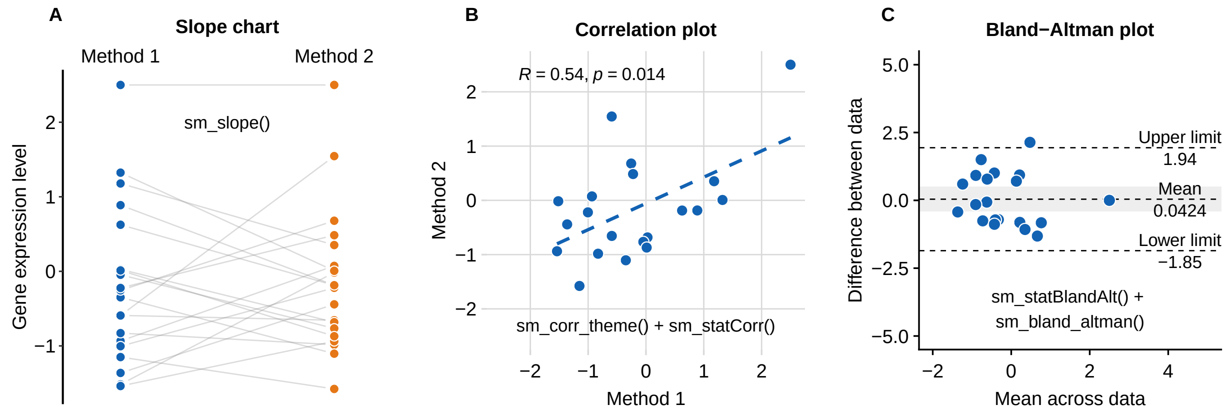


```
save_plot("case_study.png",
          case_study, ncol = 3, nrow = 1, base_asp = .95,
          dpi = 600)
```
